# Effects of introducing Xpert MTB/RIF test on multi-drug resistant tuberculosis diagnosis in KwaZulu-Natal South Africa

**DOI:** 10.1186/1471-2334-14-442

**Published:** 2014-08-16

**Authors:** Nomonde R Dlamini-Mvelase, Lise Werner, Rogerio Phili, Lindiwe P Cele, Koleka P Mlisana

**Affiliations:** Department of Medical Microbiology, University of KwaZulu-Natal, Level 4, Laboratory Building IALCH, Durban, South Africa; National Health Laboratory Services, Durban, South Africa; Centre for AIDS Programme of Research in South Africa, Durban, South Africa

**Keywords:** Xpert, Rifampicin, Resistant, MTB, South Africa

## Abstract

**Background:**

An algorithm instituted following Xpert MTB/RIF (Xpert) introduction in South Africa advocates for treating all Xpert rifampicin resistant patients as MDR-TB cases while awaiting confirmation by phenotypic or genotypic drug susceptibility testing. This study evaluates how the Xpert has influenced the diagnosis and management of drug resistant TB in the highest burdened district of KwaZulu-Natal Province.

**Methods:**

Data was retrospectively collected from all patients with rifampicin resistance on Xpert performed between March 2011 and April 2012. Xpert results were compared with those of phenotypic and/genotypic drug susceptibility testing. Patients’ records were used to determine the time to treatment initiation.

**Results:**

Out of 637 patients tested by Xpert, 50% had confirmatory results, of which a third were sent on the same day as Xpert test. The rate of rifampicin discordance and monoresistance was 8.8% and 13.4% respectively and there was no difference between phenotypic and genotypic confirmation. Among those who had been initiated on treatment, 28%, 40%, 21% and 8% of patients commenced within 2 weeks, 1 month, 2 months and 3 months of Xpert testing respectively, while the remaining 3% were observed without treatment.

**Conclusion:**

This study emphasizes the importance of complying with the algorithm in confirming all Xpert rif resistant cases so as to ensure proper management of these patients. Despite the rapidity of the Xpert results, only about 70% of patients had been initiated treatment at one month. Therefore there is a definite need to improve the health systems in order to improve on these delays.

**Electronic supplementary material:**

The online version of this article (doi:10.1186/1471-2334-14-442) contains supplementary material, which is available to authorized users.

## Background

Whilst South Africa is the third country with the highest burden of tuberculosis (TB) in the world, following India and China, it is also overburdened with an HIV prevalence of 17.9% equating to 17% of the global burden of HIV [1–3]. More than 60% of TB infected patients in South Africa are co-infected with HIV, further complicating the diagnosis and management of these two diseases. The country is ranked fifth in the world with highest drug resistant TB cases and the multi-drug resistant (MDR) TB prevalence is 1.8% among new TB cases and 6.7% among TB retreatment cases [4]. The KwaZulu-Natal (KZN) province alone which is also disproportionally afflicted with HIV and TB co-infections, carries 36% and 37% of the country’s burden of MDR and XDR-TB cases respectively [5]. It was this overwhelming burden of disease that prompted the South African Department of Health to introduce the Xpert MTB/RIF (Cepheid GeneXpert, Sunnyvale, Ca, USA) in 2011 to improve the diagnosis of TB and detection of drug resistant TB.

The introduction of the Xpert MTB/RIF (Xpert) test replaces the two sputum smears in the initial diagnoses of TB. All cases detected as having rpoB mutations on this assay are initiated on MDR TB treatment while a second sample is sent for culture and drug susceptibility testing (DST) to confirm these results [5]. This means that patients can effectively be started on MDR-TB treatment within a week instead of the long waiting periods associated with culture results, thus reducing patient loss to follow up related to long turnaround time results [6]. The mean delay in starting MDR-TB treatment has previously been 12 weeks [7] prior to the introduction of decentralised MDR-TB treatment sites which increased the capacity for earlier treatment initiation. Decentralisation in KZN resulted in an improved but still unacceptable time to treatment of 72 versus 93 days in centralised site [8].

Despite the fact that rifampicin resistance has been used as a surrogate marker for MDR-TB as more than 90% of these cases are associated with isoniazid (INH) resistance [9–11], there is a growing concern for the increasing rifampicin monoresistance in South Africa [12, 13]. This implies that if these patients were treated as having MDR-TB, a significant number would be inadvertently denied INH. Although several studies have shown a high Xpert rifampicin resistance specificity (94-100%), the positive predictive value remains low in areas with a low prevalence of MDR-TB like South Africa [14, 15], hence the need for confirmatory culture. Our guidelines for drug resistant TB require that the confirmatory sample be sent to the laboratory when the patient returns for the Xpert results. There are no studies that have assessed the frequency of return for second sampling and the extent of delay thereof.

The aim of this study was to ascertain how the introduction of Xpert has influenced the health care practices in the management of MDR-TB in KZN by determining the rate of patient return for the confirmatory culture sample, whether it could be linked to the initial GXP result and the period it takes before starting MDR-TB treatment. In addition to this, we also aim to determine the rate of rifampicin monoresistance and concordance when compared with culture.

## Methods

### Study design

The study was an observational retrospective study using data of all patients who showed rifampicin resistance on the Xpert test performed on the GeneXpert at the Prince Mshiyeni Memorial Hospital (PMMH) laboratory over one year period between March 2011 and April 2012.

### Study setting

PMMH is a public regional hospital located in the biggest township in KZN in Durban. It has 1200 beds which serve the surrounding community including 18 primary health clinics. It was selected for the placement of the GeneXpert 48 infinity due to the high burden of TB and HIV in the area. Surrounding hospitals in the sub-district also send their sputum specimens for processing at this Hospital. Patients diagnosed with drug resistant TB are further referred to a 320 beds hospital that manages drug resistant TB patients.

### Laboratory procedures

The National Health Laboratory Service (NHLS) operates on a national laboratory information system (LIS) which allows tracking of patients results as all the public laboratories utilise this LIS. The Xpert MTB/RIF test was performed according to the manufacturer’s instructions. The GeneXpert infinity is interfaced with the LIS; therefore all tests performed by the instrument are available on the system. All the data from the LIS is processed centrally by the central data warehouse (CDW). The Xpert results are printed directly at the patient facilities using SMS printers, while printed copies are distributed from the laboratory to the healthcare facilities on the next day.

The *Mycobacterium tuberculosis* (MTB) culture and drug susceptibility testing (DST) was performed at the Central Academic Laboratory using the standard laboratory procedures for culture and indirect line probe assay (LPA) [GenoType MTBDR*plus* assay, Hain Lifescience, Nehren, Germany] and/the 1% indirect agar proportion method (APM) using Middlebrook 7H10 agar. This laboratory is a quality assured reference laboratory for the KZN province. Culture for TB was performed using BACTEC mycobacteria growth indication tubes (MGIT) 960 system [BACTEC MGIT Becton Dickinson, USA]. Positive cultures were tested for susceptibility to first line TB drugs using the LPA first in order to confirm resistance to rifampicin and isoniazid. Where these results demonstrated resistance to any of the drugs tested, further testing for both first and second line drugs was performed using APM. Samples coming from patients in the MDR-TB units were tested using APM with no preceding LPA.

### Data collection and analysis

The Xpert rifampicin resistant data was obtained from the CDW and from the LIS. Demographic data was used to link Xpert results with the culture results. Patients that could not be reliably linked were regarded as untraceable and excluded from further analysis. All samples registered in the laboratory within 48 hours of each other were regarded as same day samples. Where both the LPA and APM were performed, the result of the APM was used for comparison with the Xpert result, where only LPA was done; it was used for comparison. The information containing the date of commencing MDR TB treatment was collected from the TB register at admitting Hospital. Patients were traced up to 3 months from the date of Xpert sample and cases which were not identified on the TB register after 3 months were considered as missing.

For those patients with both the Xpert and culture results, the period between the two samples was determined. Descriptive statistics were used to report proportions of patients who measured rifampicin monoresistant, MDR-TB and rifampicin susceptible on both LPA and DST tests. A Chi square test for agreement was used to compare the agreement of results between the LPA and APM diagnostic methods. The amount of time taken to commence treatment was calculated by subtracting the date of Xpert registration in the laboratory from the date of starting TB treatment. Time to treatment was categorized and described Analysis was performed using SAS version 9.3 (SAS Institute Inc., Cary) and graphs were drawn using Microsoft Excel.

The study protocol was approved by the University of KwaZulu-Natal Biomedical Research Ethics Committee.

## Results

**Figure 1:**
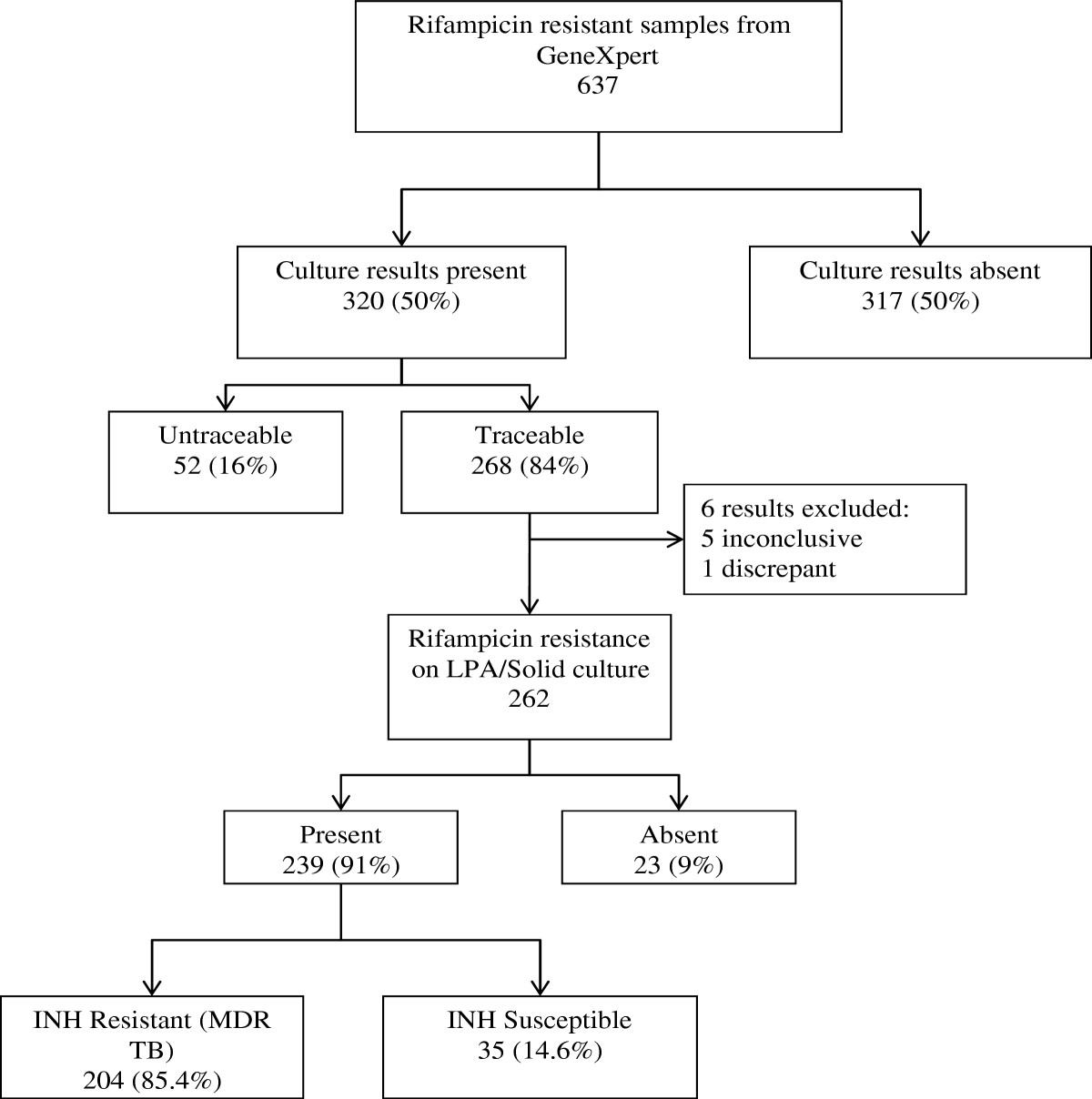
**Flow diagram of results of patients with Rifampicin resistance on Xpert.**

**Figure 2:**
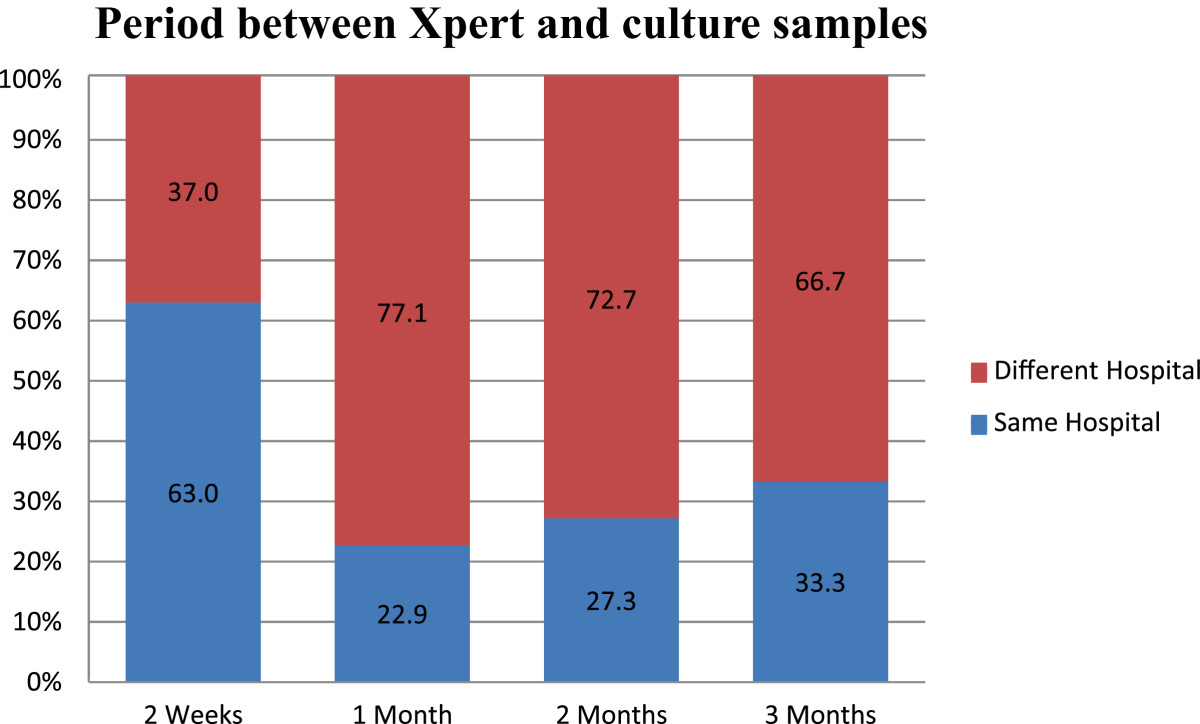
**Site and timing of collection of Xpert and Confirmatory samples.**

Out of 34 444 patients tested for TB using Xpert at PMMH laboratory between March 2011 and April 2012, 5870 were positive for TB. Of these, 637 showed rifampicin resistance but after excluding the untraceable results, only 268 (42%) had a culture sample for comparison with the Xpert and therefore eligible for further analysis (Figure 1). About a third (32.8%) of the confirmatory samples were submitted simultaneously with the Xpert samples (paired samples) with an additional 20% being sent within two weeks of Xpert (Table 1). Most of the confirmatory samples submitted within 2 weeks of Xpert (63%) were from the same hospital where Xpert sample was collected while those collected at a different hospital to that of Xpert accounted for the majority of those collected later (Figure 2).

**Table 1:** **Time between date of Xpert and culture samples**

Period between samples	Number of patients	Percentage
Culture prior to GXP	22	8.2%
Paired samples	88	32.8%
0-2 weeks	53	19.8%
>2-4 weeks	55	20.5%
>4-8 weeks	26	9.7%
>8-12 weeks	9	3.4%
>12 weeks	15	5.6%
**Total**	**268**	**100**

Most of the rifampicin resistance confirmation was performed using APM. Table 2 shows the summary of the LPA and APM results for the Xpert rifampicin resistant samples. The Xpert MTB/RIF assay showed 8.8% discordance when compared to culture and LPA/APM and the overall rate of rifampicin monoresistance was 13.4%.

**Table 2:** **Summary of Culture and LPA/APM in patients with rifampicin resistant Xpert**

Test	Rifampicin sensitive	Rifampicin monoresistant	MDR-TB	Inconclusive
Culture and LPA (N = 180)	14 (7.8%)	30 (16.7%)	130 (72.2%)	6 (3.3%)
Culture and APM (N = 226)	11 (4.9%)	31 (13.7%)	184 (81.4%)	0 (0.0%)
Total Confirmed (N = 262)	23 (8.8%;	35 (13.4%;	204 (77.8%;	N/A
	5.4-12.2%)	9.2-17.5%)	72.8-82.9%)	N/A

When comparing the LPA and APM results for the 144 samples where both were available, 87% (126) of LPA results agreed with APM (Table 3). An agreement test failed to show a significant difference in agreement between LPA and APM, however there does appear to be a trend (p = 0.062).

**Table 3:** **Comparison of LPA and APM results**

Number of samples	APM results			
LPA results	RIF susceptible	RIF monoresistant	MDR-TB	Inconclusive
RIF susceptible	**2**	0	0	0
RIF monoresistant	3	**17**	6	0
MDR-TB	1	2	**107**	0
Inconclusive	2	3	1	**0**

Boldface shows agreement between LPA and APM results.

Out of the 268 patients with confirmatory samples, 98 (36%) were not found on the MDR-TB treatment register within 3 months of Xpert samples. Of the remaining 170 patients that started treatment, 28% were started within two weeks of Xpert test, with the majority (68%) already started treatment within a month of Xpert (Table 4).

**Table 4:** **Period between Xpert samples and commencement of treatment**

Time from GXP diagnosis to treatment initiation	Number of patients	Percentage	Cumulative percentage
0-2 weeks	48	28.2	28.2%
2-4 weeks	68	40.0	68.2%
4-8 weeks	35	20.6	88.8%
8-12 weeks	14	8.2	97.0%
None	5	3.0	100%
**Total**	**170**	**100**	**100**

## Discussion

This study reveals lessons that have been learnt during the initial introduction of Xpert. Considerable gaps were identified regarding compliance with the current national guidelines in the diagnosis and management of MDR-TB using Xpert [16].

There was poor adherence to the Xpert algorithm especially with sending a confirmatory sample. Even though we could not show the reasons behind this finding, several explanations could be attributed to this non-compliance, including poor training of health care workers at the time of Xpert introduction. The rollout of Xpert preceded the training of clinicians; this may have lead to poor understanding of the role of the assay in the diagnosis of DR TB. Other reasons could be patient related, as shown in several studies conducted in South Africa where the initial defaulter rate was between 16 and 17% [17, 18]. The majority of the patients in these studies could not be interviewed in order to find reasons behind their default because they were either lost to follow up (45%), or demised prior to starting treatment (24%). In recognition of this, the provincial department of health in collaboration with the laboratory services has recently established an alert system where health care workers at patient facilities are alerted of MDR TB results. This should lead to prompt tracing of patients for further management.

A significant number of samples were submitted simultaneously for both Xpert and culture. While this may have ensured timely confirmation of Xpert rifampicin resistant cases, both tests were performed concurrently without considering the Xpert results. This practice could lead to wastage of resources where Xpert results do not require a confirmatory test. The Western Cape which is one of the provinces in South Africa has a protocol where two samples are submitted concurrently, one for Xpert and should the rifampicin be resistant, the other one is used for confirmation using LPA or APM [19]. Should this practice be adopted by the rest of the country, it could improve the confirmation of Xpert resistant cases.

Our study shows a slightly lower concordance between Xpert and culture than previously reported despite the previous assay improvements made in 2010 [20]. Several studies conducted in South Africa have shown excellent Xpert rifampicin resistance specificity of between 99.4 and 99.5% [19, 21]. However these studies used molecular assays (LPA and sequencing) which have recently been proven to show a better concordance to Xpert than the phenotypic assays [22]. Interestingly, as shown in Table 3, out of the seven patients who had discordant Xpert rifampicin resistance where both the LPA and APM were performed for confirmation, only two were sensitive to rifampicin on LPA while all were sensitive on APM. We were unable to show any significant difference between using the phenotypic or genotypic test for confirmation because of the small number of cases.

Traditional phenotypic DST is considered the gold standard for TB drug resistance testing; however studies are now showing cases of TB isolates that have rpoB mutation that are not detected by phenotypic assays [23–26]. Williamson et al. [26] reported cases with INH mono-resistance that demonstrated genotypic rifampicin resistance while phenotypically susceptible to rifampicin on proportion method using MGIT. These patients subsequently failed treatment. Almost half of our culture confirmed rifampicin susceptible (discordant) cases were resistant to INH. The isolates were not available for sequencing, but, further studies that look at the presence of these low level mutations are warranted.

The discordance of rifampicin resistance can lead to diagnostic and management dilemmas because DR-TB management has serious consequences. Patients are not only given more toxic and less effective second line drugs, but they are also subjected to socioeconomic disruption as they have to get their treatment from the designated MDR-TB treatment facilities. Given the limitations of the available laboratory diagnostic assays, discordance between Xpert and phenotypic testing require confirmation by another molecular assay. Additionally, larger studies are essential to ascertain the clinical significance of such cases.

Our findings demonstrate a high rate of rifampicin monoresistance (13.4%) and there was no statistical difference between using LPA and APM, hence both these test were reliable in confirming MDR TB. A study conducted by Coovadia and colleagues in our setting between 2007 and 2009, showed the overall rifampicin monoresistance rate of 8.8% [13]. This increasing rifampicin resistance show that rifampicin can no longer predict MDR TB in a significant number of patients, further emphasizing the importance of performing confirmatory DST. Whereas these patients might benefit from adding isoniazid on their MDR TB treatment regimen while awaiting culture and DST results, the majority of patients would be exposed to this drug unnecessarily, particularly because most of these patients are already on multiple drugs due to co-infection with HIV.

Among patients found on the TB register, 68% started treatment within one month of Xpert test. Although this is a major improvement when comparing to the pre-Xpert period [7, 27], the delay is still substantial considering the rapidity of the Xpert together with the decentralization of the MDR TB treatment. In a feasibility study conducted by Boehme *et al.*[28], Xpert reduced time to treatment in smear negative TB from 56 days to 5 days, the latter has also been advocated by the SA department of Health as the cut off period for starting MDR TB treatment. The TB/HIV care project in rural KZN was able to put 89% of patients on treatment within five days [29]. This was achieved by using Xpert for diagnosis, while the results were communicated using cell phones to link patients with community health workers, clinics and laboratory. This could be adopted by the department of health in order to improve time to treatment initiation. An efficient health care system is crucial to achieve a significant impact on drug resistant TB management.

A number of studies performed in South Africa have shown that when Xpert is done closest to the point of care, treatment can be initiated on the same day [30–32]. An average of 71% of results achieved the targeted turnaround time of 48 hours at PMMH laboratory over the past year. Lawn and colleagues showed that performing Xpert at a centralised laboratory increases the turnaround time of results by up to four days, which could impact on the initiation of treatment [14, 33]. Whilst these studies mainly involved patients with drug sensitive TB, further decentralisation of MDR TB treatment sites together with transfer of Xpert to the near point of care could bring significant improvement to these treatment initiation delays.

The number of patients that were untraceable is a cause for concern. These were patients who did not have enough details on the computer system to link the Xpert result to the culture result. This reflects poor data capturing and matching these result would erroneously cause increase in the rate of discordant result. Given that patients with MDR TB are treated in designated facilities which are different to the patient’s local facility, it becomes very important to have correct and complete patient details so as to follow up on the results. Failing to do so may results in repeated testing which not only leads to wastage but may also cause delays in changing to appropriate treatment where necessary.

There were several limitations to this study. Even though the matching of Xpert and culture results was carefully carried out and results with insufficient details were excluded from analysis, this method is not perfect; hence there may still be minor mismatching. We studied the period of the initial roll out of the Xpert; there may have been subsequent improvements in the management of MDR TB patients as the test was getting more recognised throughout the country. Finally, while the area studied is a high burden TB region, our findings may not be representative of the whole province.

## Conclusion

A few lessons have been learnt following the introduction of Xpert. Introducing a new test requires proper preparation and training of those involved in order to ensure compliance with the stipulated guidelines. Our findings show a significant amount of discordance between Xpert and phenotypic and/ genotypic drug susceptibility testing which underscores the importance of taking a second sample in cases of Xpert rifampicin resistance. This will also assist in detection of the increasing rifampicin monoresistance. Studies ascertaining the causes and clinical significance of discordance between phenotypic and genotypic assays are warranted in order to solve the diagnostic dilemmas that often accompany such results. Despite having a rapid diagnostic tool which can generate results in a few hours, system associated challenges continue to result in delays in treatment initiation.

## Authors’ original submitted files for images

Below are the links to the authors’ original submitted files for images. Authors’ original file for figure 1 Authors’ original file for figure 2

## Abbreviations

APM: Agar proportion method
CDW: Central data warehouse
DST: Drug susceptibility testing
INH: Isoniazid
KZN: KwaZulu-Natal
LIS: Laboratory information system
LPA: Line probe assay
MDR-TB: Multidrug resistant tuberculosis
MGIT: Mycobacteria growth indicator tube
NHLS: National health Laboratory Services
PMMH: Prince Mshiyeni Memorial Hospital
Xpert: Xpert MTB/RIF.

## References

[1] World Health Organization (2011). Global Tuberculosis Control.

[2] National Antenatal Sentinel HIV and Syphilis Prevalence Survey in South Africa. http://www.health.gov.za/docs/reports/2013/Antenatal_survey_report_2012_web_optimized.pdf,

[3] Abdool Karim SS, Churchyard GJ, Abdool Karim Q, Lawn SD (2009). HIV infection and tuberculosis in South Africa: an urgent need to escalate the public health response. Lancet.

[4] World Health Organization, South Africa. 2010, Available: http://www.who.int/tb/country/en/index.html, . Tuberculosis Profile

[5] Department of Health, Republic of South Africa (2011). Management of Drug-Resistant Tuberculosis: Policy Guideline.

[6] Claassens MM, du Toit E, Dunbar R, Lombard C, Enarson DA, Beyers N, Borgdorff MW (2013). Tuberculosis patients in primary care do not start treatment. What role do health system delays play?. Int J Tuberc Lung Dis.

[7] Narasimooloo R, Ross A (2012). Delay in commencing treatment for MDR TB at a specialised TB treatment centre in KwaZulu-Natal. SAMJ.

[8] Loveday M, Wallengren K, Voce A, Margot B, Reddy T, Master I, Brust J, Chaiyachati K, Padayatchi N (2012). Comparing early treatment outcomes of MDR TB in decentralised and centralised settings in KwaZulu-Natal, South Africa. Int J Tuberc Lung Dis.

[9] Somoskovi A, Parsons LM, Max SM (2001). The molecular basis of resistance to isoniazid, rifampin, and pyrazinamide in *Mycobacterium tuberculosis*. Respir Res.

[10] Pablos-Mendez A, Raviglione M, Laszlo A, Binkin N, Rieder HL, Bustreo F, Cohn DL, Lambregts-van Weezenbeek CS, Kim SJ, Chaulet P, Nunn P (1998). Global Surveillance for Antituberculosis-drug resistance, 1994–1997. NEJM.

[11] Weyer K, Brand J, Lancaster J, Levin J, van der Walt M (2007). Determinants of multidrug-resistant tuberculosis in South Africa: results from a national survey. SAMJ.

[12] Mukinda FK, Theron D, van der Spuy GD, Jacobson KR, Roscher M, Streicher EM, Musekiwa A, Coetzee GJ, Victor TC, Marais BJ, Nachega JB, Warren RM, Schaaf HS (2012). Rise in rifampicin-monoresistant tuberculosis in Western Cape, South Africa. Int J Tuberc Lung Dis.

[13] Coovadia YM, Mahomed S, Pillay M, Werner L, Mlisana K (2013). Rifampicin mono-resistance in Mycobacterium tuberculosis in KwaZulu-Natal, South Africa: a significant phenomenon in a high prevalence TB-HIV region. PLoS One.

[14] Lawn SD, Brooks SV, Kranzer K, Nicol MP, Whitelaw A, Vogt M, Bekker L, Wood R (2011). Screening for HIV-associated tuberculosis and rifampicin resistance before antiretroviral therapy. A prospective study. PLoS Med.

[15] Boehme CC, Nabeta P, Hillemann D, Nicol MP, Shenai S, Krapp F, Allen J, Tahirli R, Blakemore R, Rustomjee R, Milovic A, Jones M, O’Brien SM, Persing DH, Ruesch-Gerdes S, Gotuzzo E, Rodrigues C, Alland D, Perkins MD (2010). Rapid molecular detection of tuberculosis and rifampin resistance. NEJM.

[16] GeneXpert Implementation in South Africa Public Sector. 2012, http://webcache.googleusercontent.com/search?q=cache:crqSYvd3y_AJ:www.stoptb.org/wg/gli/assets/html/day%25203/Stevens%2520-%2520South%2520Africa.pdf+&cd=1&hl=en&ct=clnk&gl=za,

[17] Botha E, den Boon S, Lawrence KA, Reuter H, Verver S, Lombard CJ, Dye C, Enarson DA, Beyers N (2008). From suspect to patient: tuberculosis diagnosis and treatment initiation in health facilities in South Africa. Int J Tuberc Lung Dis.

[18] Botha E, Den Boon S, Verver S, Dunbar R, Lawrence KA, Bosman M, Enarson DA, Toms I, Beyers N (2008). Initial default from tuberculosis treatment: how often does it happen and what are the reasons?. Int J Tuberc Lung Dis.

[19] Osman M, Simpson JA, Caldwell J, Bosman M, Nicol MP (2014). GeneXpert MTB/RIF version G4 for identification of rifampin-resistant tuberculosis in a programmatic setting. J Clin Microbiol.

[20] Steingart KR, Sohn H, Schiller I, Kloda LA, Boehme CC, Pai M, Dendukuri N (2013). Xpert® MTB/RIF assay for pulmonary tuberculosis and rifampicin resistance in adults. Cochrane Database Syst Rev.

[21] Theron G, Peter J, van Zyl-Smit R, Mishra H, Streicher E, Murray S, Dawson R, Whitelaw A, Hoelscher M, Sharma S, Pai M, Warren R, Dheda K (2011). Evaluation of the Xpert MTB/RIF assay for the diagnosis of pulmonary tuberculosis in a high HIV prevalence setting. Am J Respir Crit Care.

[22] Rigouts L, Gumusboga M, de Rijk WB, Nduwamahoro E, Uwizeye C, de Jong B, Van Deun A (2013). Rifampin resistance missed in automated liquid culture system for *Mycobacterium tuberculosis* Isolates with Specific *rpoB* mutations. J Clin Microbiol.

[23] Van Deun A, Barrera L, Bastian I, Fattorini L, Hoffmann H, Kam KM, Rigouts L, Wright A, Ru¨sch-Gerdes S (2009). Mycobacterium tuberculosis strains with highly discordant rifampin susceptibility test results. J Clin Microbiol.

[24] Ho J, Jelfs P, Sintchencko V (2013). Phenotypically occult multidrug-resistant Mycobacterium tuberculosis: dilemmas in diagnosis and treatment. J Antimicrob Chemother.

[25] Somoskovi A, Deggim V, Ciardo D, Bloemberg GV (2013). Diagnostic implications of inconsistent results obtained with the Xpert MTB/Rif assay in detection of Mycobacterium tuberculosis isolates with an rpoB mutation associated with low-level rifampin resistance. J Clin Microbiol.

[26] Williamson DA, Roberts SA, Bower JE, Vaughan R, Newton S, Lowe O, Lewis CA, Freeman JT (2011). Clinical failures associated with rpoB mutations in phenotypically occult multidrug-resistant Mycobacterium *tuberculosis*. Int J Tuberc Lung Dis.

[27] Bamford CM, Taljaard JJ (2010). Potential for nosocomial transmission of multidrug-resistant (MDR) tuberculosis in a South African tertiary hospital. SAMJ.

[28] Boehme CC, Nicol MP, Nabeta P, Michael JS, Gotuzzo E, Tahirli R, Gler MT, Blakemore R, Worodria W, Gray C, Huang L, Caceres T, Mehdiyev R, Raymond L, Whitelaw A, Sagadevan K, Alexander H, Albert H, Cobelens F, Cox H, Alland D, Perkins MD (2011). Feasibility, diagnostic accuracy, and effectiveness of decentralised use of the Xpert MTB/RIF test for diagnosis of tuberculosis and multidrug resistance: a multicentre implementation study. Lancet.

[29] TB/HIV Care Association South Africa-STOP TB Partnership. Available: http://www.stoptb.org/assets/documents/global/awards/tbreach/SouthAfrica%20TBHIVCA.pdf

[30] Hanrahan CF, Selibas K, Deery CB, Dansey H, Clouse K, Bassett J, Scott L, Stevens W, Sanne I, Van Rie A (2013). Time to treatment and patient outcomes among TB suspects screened by a single point-of-care Xpert MTB/RIF at a primary care clinic in Johannesburg, South Africa. PLoS One.

[31] Van Rie A, Page-Shipp L, Hanrahan CF, Schnippel K, Dansey H, Bassett J, Clouse K, Scott L, Stevens W, Sanne I (2013). Point-of-care Xpert MTB/RIF for smear negative tuberculosis suspects at a primary care clinic in South Africa. Int J Tuberc Lung Dis.

[32] Theron G, Zijenah L, Chanda D, Clowes P, Rachow A, Lesosky M, Bara W, Mungofa S, Pai M, Hoelscher M, Dowdy D, Pym A, Mwaba P, Mason P, Peter J, Dheda K (2014). Feasibility, accuracy, and clinical effect of point-of-care Xpert MTB/RIF testing for tuberculosis in primary-care settings in Africa: a multicentre, randomised, controlled trial. Lancet.

[33] Lawn SD, Kerkhoff AD, Wood R (2012). Location of Xpert MTB/RIF in centralised laboratories in South Africa undermines potential impact. Int J Tuberc Lung Dis.

[CR34] The pre-publication history for this paper can be accessed here:http://www.biomedcentral.com/1471-2334/14/442/prepub

