# Supplementary figures and images for: Effects of introducing Xpert MTB/RIF test on multi-drug resistant tuberculosis diagnosis in KwaZulu-Natal South Africa
Source: BMC Infect Dis. 2014 Aug 16;14:442. doi: 10.1186/1471-2334-14-442 (PMC4141089; doi:10.1186/1471-2334-14-442)

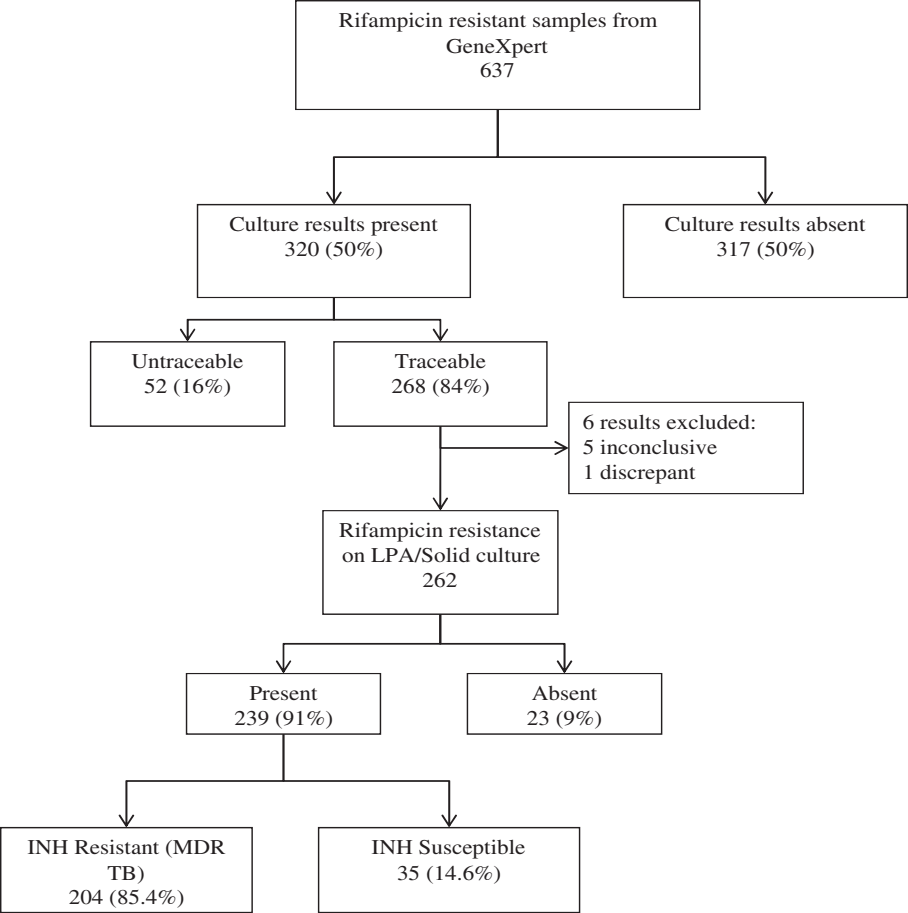

Supplement: Supplementary file 1 — Authors’ original file for figure 1 [file 12879_2014_3741_MOESM1_ESM.pdf]

# Period between Xpert and culture samples

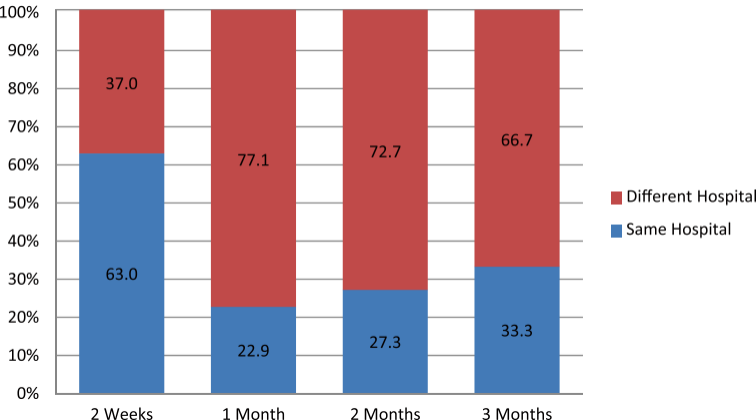

Supplement: Supplementary file 2 — Authors’ original file for figure 2 [file 12879_2014_3741_MOESM2_ESM.pdf]
